# Cardiovascular health assessed using Life’s Essential 8 is associated with all-cause and cardiovascular disease mortality among community-dwelling older men and women in the InCHIANTI study

**DOI:** 10.3389/fpubh.2025.1570463

**Published:** 2025-05-30

**Authors:** Vivek Sarma, Yichen Jin, Stefania Bandinelli, Sameera A. Talegawkar, Luigi Ferrucci, Toshiko Tanaka

**Affiliations:** ^1^School of Medicine and Health Sciences, The George Washington University, Washington, DC, United States; ^2^Department of Exercise and Nutrition Sciences, Milken Institute School of Public Health, The George Washington University, Washington, DC, United States; ^3^Geriatric Unit, Azienda Sanitaria Firenze (ASF), Florence, Italy; ^4^Longitudinal Study Section, Translational Gerontology Branch, National Institute on Aging, Baltimore, MD, United States

**Keywords:** mortality, cardiovascular disease, cardiovascular health, Life’s Essential 8, older adults, InCHIANTI

## Abstract

**Introduction:**

The older population is growing fast, and it is important to investigate the cardiovascular health factors and behaviors that are associated with cardiovascular disease (CVD) and mortality among older individuals.

**Methods:**

A total of 920 older adults (mean age: 74 years, 55% women) from the InCHIANTI study were included for analysis. Cardiovascular health (CVH) was assessed using the Life’s Essential 8 (LE8) developed by the American Heart Association, including 8 health behaviors (smoking, diet, physical activity, sleep) and factors (body mass index, blood cholesterol, blood glucose, blood pressure). The LE8 score ranges from 0 to 100, with higher score indicating better CVH. CVH was analyzed on both a continuous scale [per 1 standard deviation (SD) increase] and categorical scale [low (CVH) score < 50 vs. moderate/high (CVH) score ≥ 50]. Cox hazard models were used to calculate the hazard ratios (HRs) of CVD and all-cause mortality associated with CVH and its components adjusting for age, sex, study site, education, presence of ADL and IADL limitations, cognitive impairment, depression, and presence of chronic disease.

**Results:**

The median follow-up time was 14.5 years. Participants with moderate/high CVH had better survival rates for both CVD and all-cause mortality compared to those with low CVH. One SD increase in LE8 score was associated with 28% (*p* = 0.001) and 17% (*p* < 0.001) lower risk of CVD and all-cause mortality, respectively.

**Discussion:**

Among older community dwelling men and women, better CVH is inversely associated with CVD and all-cause mortality, and this lends credence to the importance of prioritizing health factors and behaviors in preventing chronic disease and promoting healthier lives among older adults.

## 1 Introduction

The worldwide population of older individuals has grown exponentially in the past few years and is projected to have a rapidly increasing trajectory for high-income countries (1, 2). Factors responsible for this phenomenon include improvements in health care and the subsequent reduction in the severity of disability and neurodegenerative conditions (3). However, this extension in the life-span is also associated with decrease in the health-span, posing concerns for complex care management and healthcare costs (4).

Health factors—elevated blood pressure and glucose levels and high cholesterol—as well as adverse health behaviors including smoking, poor diet, and insufficient physical activity, each elevate an individual’s risk for chronic disease and mortality (5–11). Life’s Simple 7 (LS7), an American Heart Association (AHA)-developed metric highlighting the role of each of these factors and behaviors in chronic disease development, has been evaluated in prior studies as a framework for assessing an individual’s clinical risk of mortality due to chronic disease (12). In a previous study (13), we modeled cardiovascular health (CVH) by incorporating each health behavior and factor outlined in LS7 along with the overall score and found that higher LS7 scores (indicating more robust CVH) corresponded to reduced mortality due to cardiovascular disease (CVD) and overall.

In our newer study, we examined Life’s Essential 8 (LE8), an updated rendering of LS7 that incorporates sleep as an additional factor predictive of chronic disease risk (14). We derived a single quantitative CVH score incorporating all 8 behaviors (diet, physical activity, tobacco smoking, sleep) and factors (body mass index, blood pressure, serum glucose, and cholesterol levels) outlined in this newer framework and evaluated the relationship between scores and mortality from cardiovascular and all causes in a cohort of community-dwelling men and women aged 65 years and older. This final score—termed “LE8 score”—serves as a comprehensive assessment of a study participant’s overall CVH. Further, we hypothesized that among cohort participants, higher LE8 scores (indicating better CVH) correspond to reduced risk of all-cause mortality and mortality from CVD.

## 2 Materials and methods

The InCHIANTI study is a prospective population-based study of aging, integrating participants from two cities: Greve in Chianti and Bagno a Ripoli in Tuscany, Italy (15). There were 1,453 participants that were recruited during 1998 to 2000 using a two-stage stratified sampling procedure and were then followed longitudinally. Every 3 years, data on various study variables were collected through home interview, medical exam, and a clinical exam at the study site. We excluded participants missing data on any LE8 components (*n* = 241), those reporting implausible energy intakes (< 600 or > 4,000 kcal/day, *n* = 7), younger than 65 years of age (*n* = 267), or who were diagnosed with dementia at baseline (*n* = 18) to reduce inaccurate responses to the FFQ, at baseline. The final analytic sample size was 920 (Supplementary Figure 1). The study protocol was approved by the Italian National Institute of Research and written informed consent was obtained from participants at each visit.

### 2.1 Overall cardiovascular health

Supplementary Table 1 displays each component’s scoring criteria. To assess a participant’s dietary intake, a food frequency questionnaire was utilized which allowed participants of the InCHIANTI study to self-report their diets. Subsequently, the Mellen’s score—ranging from 0 to 9—was computed for participants to assess their dietary quality and adherence to the Dietary Approaches to Stop Hypertension (DASH) diet. To assess physical activity, self-report over the past year was used. Smoking status was assessed solely based on cigarette usage, as data on secondhand smoke exposure and usage of inhaled nicotine delivery systems was unavailable. Duration of sleep was obtained through a standard survey.

Measured weight (kg) and height (m) were used to compute BMI. Blood pressure of participants was measured using a mercury sphygmomanometer, with participants in a supine position. There were 2-min intervals between each of the three readings for each participant. Information regarding the use of blood pressure medication was obtained through interviews.

After fasting for at least 8 h, participants provided serum samples which allowed us to assess total cholesterol and serum glucose. No HbA1c data were collected in the InCHIANTI study; hence, we used average fasting glucose levels information and a conversion chart from the American Diabetes Association to calculate the scores.^1^ Information on medication use for diabetes or elevated blood lipids was obtained through interviews.

### 2.2 Vitality ascertainment

Per the cohort protocol, all study participants were interviewed for follow-up purposes every 3 years. Alive v. dead status was verified with the Tuscany Region Mortality General Registry, as well as death certificates from the municipalities in which participants lived. Data were collected from 1998 to 2020, and the cause of death was identified using the International Classification of Diseases, Ninth and Tenth Revision (ICD-9 and 10). CVD mortality was defined as ICD-9 codes 390–459 and ICD-10 codes I00-I99.

### 2.3 Covariates

Based on our univariate analysis and previously published literature, we included several covariates in our analysis. These include age, sex, study site (Greve vs. Bagno a Ripoli), disability and cognitive statuses, years of education, depression status, and the presence of chronic disease. The criteria for scoring each component of LE8 is noted in Supplementary Table 1. The disability status of participants was assessed by evaluating abilities to perform Activities and Instrumental Activities of Daily Living (ADL and IADL). Participants were evaluated based on the distinct number of ADL and IADL tasks that could not be performed. If this number was greater than 0, participants were considered to have a disability. In regard to cognitive status, the Mini-Mental State Examination (MMSE) was used, and MMSE scores < 24 were considered to be cognitive impairment (16). The Center for Epidemiological Studies–Depression (CES-D) scale was used to evaluate depression, which was defined as a CES-D ≥ 20 (17). Chronic diseases—including heart failure, coronary disease, peripheral arterial disease, lung disease, cancer, stroke, hip arthritis, liver disease, renal disease, gastrointestinal disease, Parkinson’s disease—was framed as the presence or absence of any of these diseases.

### 2.4 Statistical analysis

CVH was analyzed on both a continuous scale [per 1 standard deviation (SD) increase] and categorical scale. The categorical scale, originally developed by the AHA, assigns a score between 0 and 100 for each category of LE8. The overall LE8 score for each participant is the average of the individual scores of each category. Scores ranging between 0–49, 50–79, and 80–100, translate to low, moderate, and high LE8 scores. In our analysis, we integrated moderate and high LE8 scores together due to the relatively small proportion of participants with a high LE8 score, so we also conducted a sensitivity analysis using tertiles of LE8 score. As baseline cardiovascular disease could affect the associations between LE8 and mortality, we conducted a second sensitivity analysis removing those with cardiovascular disease at baseline. Further, sociodemographic characteristics at baseline were reported as means (SD) or n (%). Baseline differences in sociodemographic characteristics between low and moderate/high LE8 were tested using *t*-test and chi-square tests for continuous and categorical variables, respectively. Cox proportional hazards models were implemented to examine the associations between CVH and its components with all-cause and CVD mortality. Models were adjusted for age, sex, study site, education, presence of ADL and IADL limitations, cognitive impairment, depression, and presence of chronic disease. The proportional hazards assumption was ensured by using Schoenfeld residuals test. Further, the incidence of death was calculated per 1,000 person-years. The Kaplan–Meier curve was plotted for cumulative survival probabilities. All analyses were performed using R version 4.1.3.

## 3 Results

The baseline sociodemographic and health characteristics are described in Table 1. The mean age (SD) of the cohort was 74 (6.6) years, with 55% being women. There was no demonstrated age difference between those reporting low LE8 scores as opposed to moderate or high LE8 scores. Participants with higher LE8 scores were less likely to report difficulties with ADL (*p* < 0.001) and IADL (*p* = 0.03) than their counterparts with low scores. Further, those with higher scores were less likely to report having chronic disease (*p* = 0.012), or to have died from all causes (p < 0.001) or CVD specifically (*p* = 0.02) over the follow-up period.

**Table 1:** Baseline sociodemographic and health characteristics [mean (standard deviation) or percentage] by category of overall LE8 score among InCHIANTI study participants aged 65 years and older.

Baseline characteristics	Overall	Low CVH	Moderate/High CVH	*p*-value
N	920	167	753	
Age (years)	73.9 (6.6)	73.6 (6.2)	74.0 (6.6)	0.481
Women (%)	506 (55.0)	91 (54.5)	415 (55.1)	0.952
Education (years)	5.5 (3.2)	5.5 (3.2)	5.5 (3.2)	0.919
Study Site (Bagno a Ripoli)	483 (52.5)	84 (50.3)	399 (53.0)	0.587
ADL disability^1^	32 (3.5)	14 (8.4)	18 (2.4)	< 0.001
IADL disability^2^	174 (18.9)	42 (25.1)	132 (17.5)	0.030
Cognitive impairment^3^	212 (23.0)	35 (21.0)	177 (23.5)	0.545
Depression^4^	184 (20.0)	44 (26.3)	140 (18.6)	0.031
Presence of chronic disease^5^	569 (61.8)	118 (70.7)	451 (59.9)	0.012
All-cause death	610 (66.3)	131 (78.4)	479 (63.6)	< 0.001
CVD death	225 (24.5)	53 (31.7)	172 (22.8)	0.020

1 ADL disability was assessed using the ADL questionnaire, which includes six functional tasks: bathing, dressing, toileting, getting into and out of bed, continence, and eating. Disability level was evaluated based on the number of tasks a participant could not complete.

2 IADL disability was assessed using the IADL questionnaire, which includes eight functional tasks: using the telephone, shopping, preparation of food, laundry, transportation, doing light housework, managing finances, and adhering to medications. Disability level was evaluated based on the number of tasks a participant could not complete.

3 Cognitive status was evaluated using the Mini-Mental Status Examination (MMSE). A score < 24 indicated impaired cognition.

4 Depression status was evaluated using the Center for Epidemiologic Studies-Depression (CES-D) scale. A score ≥ 16 indicated depression.

5 The presence of chronic disease (e.g., malignancy, coronary heart disease, heart failure, stroke, chronic lung disease, liver disease, renal disease, GI disease, peripheral arterial disease, hip arthritis, and Parkinson’s) was assessed based on criteria implemented in the Women’s Health and Aging Study.

**Figure 1:**
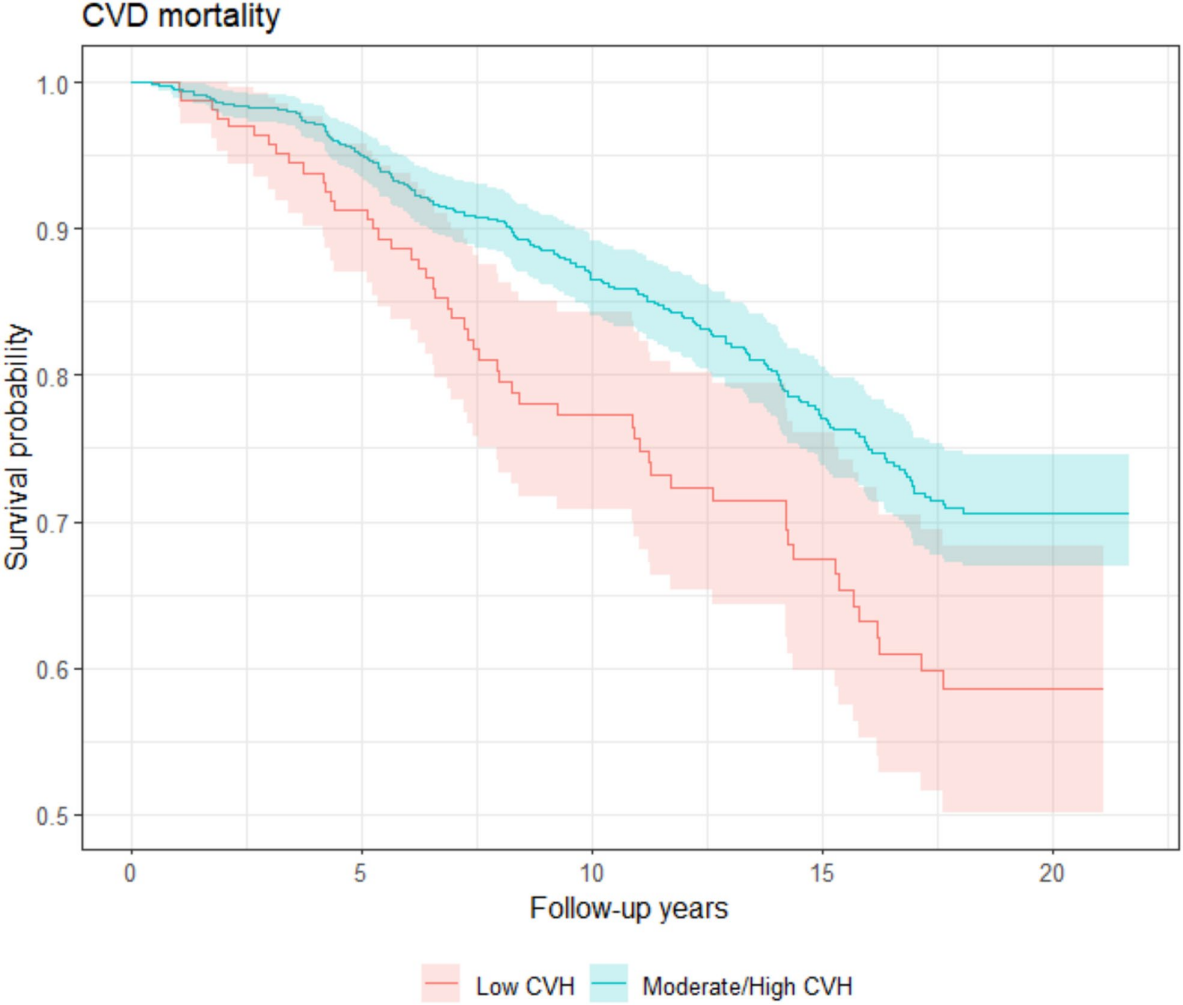
CVD-mortality Kaplan–Meier survival estimates during median follow-up of 14.5 years among InCHIANTI participants aged 65 years and older with low and moderate/high LE8 scores.

**Figure 2:**
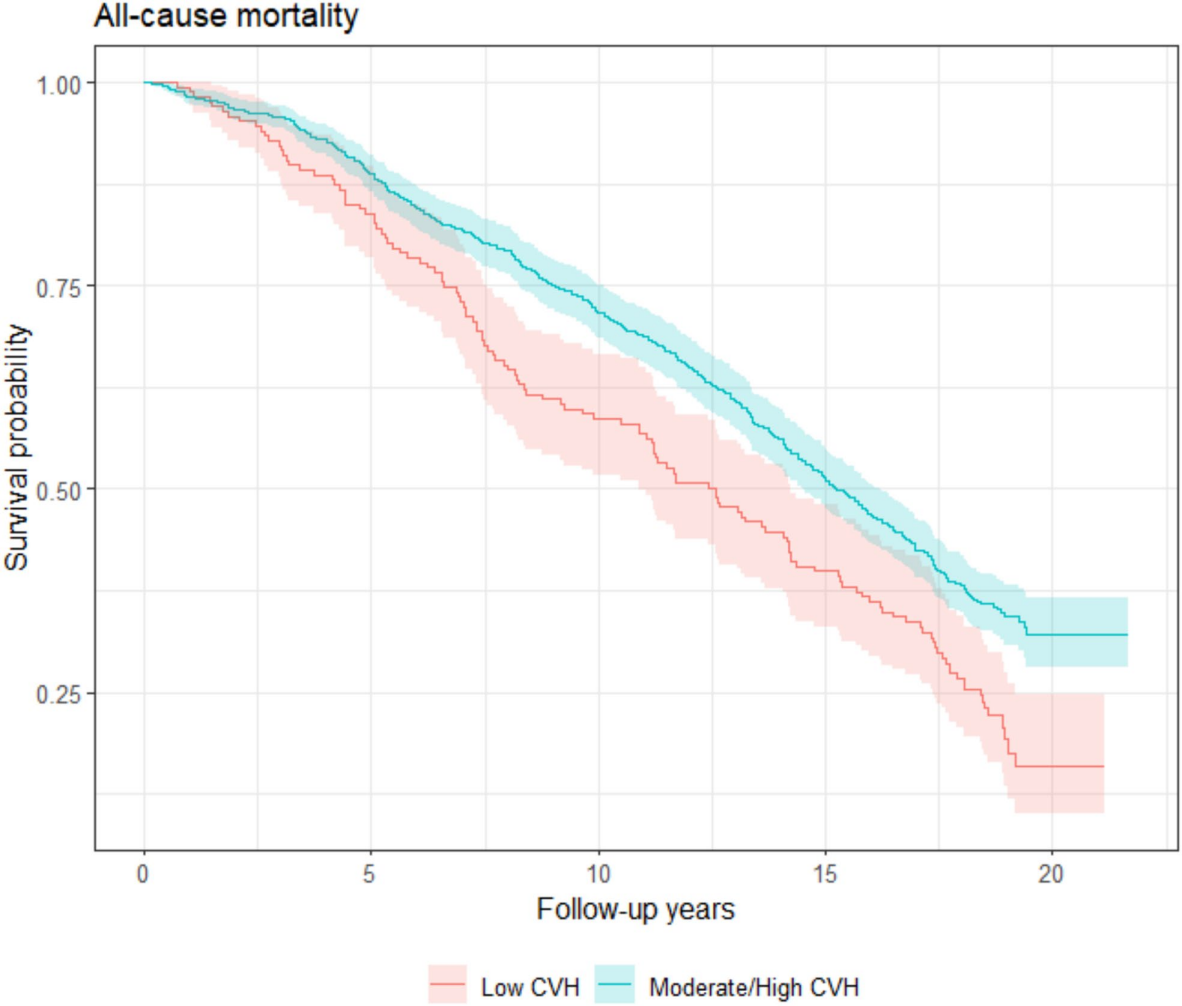
All-cause mortality Kaplan–Meier survival estimates during median follow-up of 14.5 years among InCHIANTI participants aged 65 years and older with low and moderate/high LE8 scores.

The average follow-up time for the cohort was 14.5 years. By 2020, a total of 610 people had died from all causes and 225 people had died from CVD. The incidence rate of all-cause death was 50.5 per 1,000 person-years (95% confident interval [CI]: 46.6–54.7); the incidence rate of CVD death was 18.6 per 1,000 person-years (95% CI: 16.3–21.2). Higher survival rates were reported for participants with moderate/high LE8 score compared to those with low LE8 score for both CVD and all-cause mortality according to the Kaplan–Meier survival curve (Figures 1, 2). Participants in 2nd and 3rd LE8 tertiles were also associated with lower likelihood of CVD deaths compared to those in the lowest tertile (2nd tertile: HR = 0.69, *p* = 0.020, 95% CI = 0.51–0.94; 3rd tertile: HR = 0.60, *p* = 0.004, 95% CI = 0.42–0.85) and all-cause mortality (2nd tertile: HR = 0.79, *p* = 0.014, 95% CI = 0.65–0.95; 3rd tertile: HR = 0.75, *p* = 0.007, 95% CI = 0.61–0.93). When participants with cardiovascular disease at baseline were excluded, the association between LE8 with CVD deaths (HR = 0.78 *p* < 0.001, 95% CI = 0.68–0.90) and all-cause mortality (HR = 0.83, *p* < 0.001, 95% CI = 0.77–0.91) remained significant.

When examining the individual components, there were several salient factors and behaviors that were noted to confer protection from CVD mortality (Table 2). As previously noted, a higher score for a particular health behavior or factor indicates closer adherence to recommendations. In our analysis, higher scores for the blood pressure component were associated with a lower likelihood of mortality due to CVD (HR = 0.67, *p* < 0.001, 95% CI = 0.55–0.80). In addition, a higher score for fasting serum glucose component was associated with a lower likelihood of mortality due to CVD (HR = 0.85, *p* = 0.014, 95% CI = 0.75–0.97), but the association was attenuated when adjusting for other components. Further, increases in physical activity component scores had a lower likelihood of mortality from CVD (HR = 0.77, *p* = 0.001, 95% CI = 0.65–0.90).

**Table 2:** Adjusted hazard ratio of CVD mortality for LE8 and its components among InCHIANTI participants aged 65 years and older^1^.

Score	Hazard ratio	Confidence interval	*P*-value
Overall LE8 score	0.78	0.68, 0.90	0.001
LE8 behavior	0.98	0.85, 1.14	0.821
LE8 factor	0.78	0.68, 0.89	< 0.001
LE8 components			
Diet	1.09	0.96, 1.25	0.187
Physical activity	0.77	0.65, 0.90	0.001
Smoking	0.98	0.83, 1.16	0.857
Sleep	0.91	0.79, 1.03	0.138
BMI	0.91	0.80, 1.04	0.180
Cholesterol	0.95	0.83, 1.09	0.450
Blood glucose	0.85	0.75, 0.97	0.014
Blood pressure	0.67	0.55, 0.80	< 0.001

1 This table presents the CVD mortality hazard ratios and 95% confidence intervals from Cox proportional hazard models that controlled for age, sex, study site, education, presence of ADL and IADL limitations, cognitive impairment, depression, and presence of chronic disease.

For all-cause mortality, higher scores for physical activity component (HR = 0.82, *p* < 0.001, 95% CI = 0.75–0.90) were associated with a lower likelihood of mortality (Table 3). In addition, higher scores regarding smoking status component were associated with a lower risk of death (HR = 0.8, p < 0.001, 95% CI = 0.74–0.87). Among health factors, higher scores for the serum glucose (HR = 0.91, *p* = 0.013, 95% CI = 0.84–0.98) and blood pressure components (HR = 0.87, *p* = 0.002, 95% CI = 0.79–0.95) conferred protection from all-cause death. After adjusting all components, cholesterol component score showed positively significant association with all-cause mortality (HR = 1.10, *p* = 0.020, 95% CI = 1.02–1.20).

**Table 3:** Adjusted hazard ratio of all-cause mortality for LE8 and its components among InCHIANTI participants aged 65 years and older^1^.

Score	Hazard ratio	Confidence interval	*P*-value
Overall LE8 score	0.83	0.77, 0.91	< 0.001
LE8 behavior	0.86	0.79, 0.93	< 0.001
LE8 factor	0.91	0.84, 0.99	0.021
LE8 components			
Diet	1.06	0.98, 1.15	0.135
Physical activity	0.82	0.75, 0.90	< 0.001
Smoking	0.80	0.74, 0.87	< 0.001
Sleep	0.95	0.88, 1.03	0.224
BMI	0.92	0.85, 1.00	0.054
Cholesterol	1.08	0.99, 1.17	0.080
Blood glucose	0.91	0.84, 0.98	0.013
Blood pressure	0.87	0.79, 0.95	0.002

1 This table presents the all-cause mortality hazard ratios and 95% confidence intervals from Cox proportional hazard models that controlled for age, sex, study site, education, presence of ADL and IADL limitations, cognitive impairment, depression, and presence of chronic disease.

## 4 Discussion

The objective of this study was to assess the suitability of AHA’s LE8 guidelines as a framework for determining an individual’s risk of mortality from CVD and all causes among community-dwelling individuals aged 65 years and over. The quantitative CVH assessment that we derived from LE8 incorporated several health factors (serum glucose, blood cholesterol, blood pressure, BMI) and behaviors (diet, physical activity, smoking status, sleep habits). Further, we assessed the relationship between the individual factors and behaviors that comprise LE8 and risk of mortality. Our results provide compelling evidence that, among older adults, better CVH assessed using the LE8 metric is negatively associated with risk of mortality. A higher LE8 score (indicating more robust CVH) reduces one’s risk of all-cause and CVD mortality. When examining CVH components, higher scores for physical activity, serum glucose, blood pressure, and smoking status components were associated with a lower likelihood of CVD and all-cause mortality. Notably, participants with moderate or high blood pressure component scores were seen as over 30 percent less likely to succumb to CVD mortality during follow-up. Alongside, those with moderate or high physical activity component scores were almost 25 percent less likely to pass away from cardiovascular-related mortality at any time during follow-up. Further, during follow-up, participants with moderate or high scores in smoking status were approximately 20 percent less likely to die from all causes.

Several studies have examined the relationship between CVH and mortality through the lens of the LE8 framework. A recent study of the relationship between LE8 scores and CVD mortality among middle-aged Finnish men categorized LE8 scores into quartiles (18). The results of this study found a 60% reduction in the risk of CVD mortality for those in the highest quartile compared with individuals within the lowest quartile. While this finding parallels our results, our study population was more diverse and encompassed mortality from all causes alongside CVD mortality. By investigating all-cause mortality, we had the opportunity to explore possible pathways between CVD and death from the broad spectrum of chronic disease. A study of nonpregnant, noninstitutionalized individuals between 20 and 79 years of age (19) assessed CVH in terms of its effect on life expectancy at the age of 50. Similarly to our study, LE8 scores were calculated through both categorical and continuous scales. The results of this study indicated that participants with high LE8 scores were, on average, likely to have increased their life expectancy by approximately 9 years, in comparison to those with low LE8 scores. Further, life expectancy was seen to increase approximately 2 years for every 10-point increment in LE8 score. Using data from the ENRICA study, Spanish participants aged 18 years and older in higher quartile of LE8 were associated with lower risk of all-cause and CVD deaths compared to those in the lowest quartile LE8 (20). Another study utilizing National Health and Nutrition Examination Survey (NHANES) data on individuals aged 20 years and older affirms our results (21). Similarly, LE8 scores were calculated on a scale from 0 to 100 and categorized as “low,” “moderate,” and “high.” Compared with participants with a low LE8 score, those with a high LE8 score experienced 40 and 54% reductions in the risk of all-cause and cardiovascular mortality, respectively, and similar results were reported in another study using NHANES data among 30 years and older adults (22). The study using data from Framingham Heart Study included middle-aged Framingham Offspring participants also demonstrated that those maintaining low LE8 were associated highest risk of death (23). The prior studies encompass a broad age range of participants in their assessment of the relationship between LE8 scores and mortality risk. However, our study focuses specifically on the older-aged population. It is generally believed that CVH exhibits a strong influence on the progression of chronic disease (24). In addition, chronic disease accounts for a large burden of mortality (25). As the world’s population of older-aged individuals climbs steeply, our study’s population allows us to more closely examine the interaction between CVH and chronic disease, and this interaction’s combined effect on mortality.

The community of older-aged individuals is markedly susceptible to multi-organ disease development largely due to the process of aging. As such, we felt that our assessment of both health behaviors and factors and their impact on mortality due to both cardiovascular and all-cause disease would allow us to explore possible relationships between risk factors and behaviors, CVD and disease due to other causes, the components of LE8 and all-cause disease, and age and disease mortality. In our study, increments in smoking status revealed significant impacts in the likelihood of mortality. For instance, tobacco usage is known as a distinct risk factor in the development of elevated blood pressure (26); both are individual components of LE8. Studies have also shown a correlation between tobacco smoking and dietary quality, with smokers more likely to consume processed foods and meat with inadequate intake of fiber-rich foods, such as fruits and vegetables (27). Smoking is also a distinct risk factor associated with an increased risk of diabetes and renal disease (28, 29). As such, it is plausible that the significant reduction in CVD mortality associated with a higher score of blood pressure can be attributed to separate impacts from smoking status, fasting serum glucose levels, and the presence of other diseases. Further, participants who engage in physical activity could be more likely to take an active interest in their health. As such, those with higher physical activity scores may be more likely to have higher scores in other categories of LE8. This would account for the significant decrease in the likelihood of death due to both cardiovascular and all-cause disease seen in association with physical activity in our study. Moreover, the positive association between cholesterol component and all-cause mortality is consistent with previous studies that reported higher cholesterol levels associated with lower risk of all-cause mortality and non-CVD mortality (30).

When assessing the health behaviors and risk factors of study participants, it is imperative to acknowledge the wide disparity in the opportunity to take agency over one’s health. In particular, the protective measures that improve health and wellbeing can be challenging to accomplish due to circumstances beyond one’s control. For instance, for those individuals living in industrialized environments with high population densities, a healthy exercise routine may be difficult to accomplish due to space constraints and environmentally mediated risks to health. Further, nutritious foods known to improve health—particularly those high in fiber and lower in saturated fat—are known to be more expensive, disabling individuals of lower-income communities from purchasing them (31). As such, a wide array of social determinants of health significantly impairs the potential of individuals from achieving robust health and curbing disease mortality.

Overall, our study had several strengths. We looked beyond comparing the number of added years of life to explore the likelihood of mortality and how it is influenced by variations in CVH. In addition, we modified several of the LE8 categories—smoking status, serum glucose concentration, physical activity—to better suit our study cohort and more effectively examine the effects of these behaviors and factors on mortality. The original LE8 recommendation was to utilize thresholds created by the NHANES to calculate the diet score categories. However, given the data availability and considering that the participants in our analysis are older-aged adults from rural Italy, we contended it would be more pragmatic to utilize the thresholds from the InCHIANTI study to better capture the actual diet quality of these individuals. Further, InCHIANTI is a distinctive study of well-characterized older adults that enabled us to examine the effects of health behaviors on lifespan in this demographic group. By using this representative sample of older adults, we could examine mortality from multiple lenses rather than solely focus on mortality from CVD. As this population of individuals is markedly susceptible to the wide spectrum of disease due to aging, we sought to determine the effects of each of LE8’s components on death from all causes. This allowed us to distinguish between the effect of these components on all-cause death and solely from cardiovascular-associated death.

There were several limitations to our study. Data regarding health behaviors were obtained via self-reported questionnaires, instilling the possibility of recall and social desirability biases. In turn, this may have affected the accuracy in our estimation of the effects of LE8’s individual components on mortality likelihoods by introducing measurement error and misclassification. Also, although we included most of the important confounders, some other unmeasured variables were not available to be adjusted resulting in residual confounding, which may further influence the magnitude of estimation for the associations between CVH and mortality. In addition, we only used baseline LE8 scores in our assessment of the relationship between CVH and mortality. As changes in CVH over time are feasible, a singular baseline score does not adequately reflect the trajectory in health of our study participants.

In our prior study, we derived a CVH metric based upon guidelines imposed in LS7, the former iteration of LE8. This metric was divided into tertiles, and our findings demonstrated that a score in a higher tertile (indicating the most robust CVH) was associated with a risk reduction in CVD and all-cause mortality among older adults, community-dwelling individuals. Our assessment of LE8 further strengthens the notion that the health behaviors and risk factors implicated in this framework contribute to one’s overall mortality risk due to CVD and all-cause disease. Our findings point to the importance of prioritizing health factors and behaviors in preventing chronic disease development and promoting healthier lives among older individuals. This reflects the crucial need for policy that promotes the channeling of economic resources to improve access to affordable nutrition for community-dwelling individuals such as meal delivery programs, implementation of health and nutrition education at healthcare or community centers, developing social support programs for healthy aging support. And additionally consider development of and improved access to environments safe for engaging in exercise that together promotes overall health.

## Data Availability

The raw data supporting the conclusions of this article will be made available by the authors, without undue reservation.

## Footnotes

^1^ https://professional.diabetes.org/diapro/glucose_calc
