# Supplementary material for: Cardiovascular health assessed using Life’s Essential 8 is associated with all-cause and cardiovascular disease mortality among community-dwelling older men and women in the InCHIANTI study
Source: Front Public Health. 2025 May 30;13:1570463. doi: 10.3389/fpubh.2025.1570463 (PMC12162959; doi:10.3389/fpubh.2025.1570463)
Supplement: Supplementary file 1 [file Table_1.docx]

**
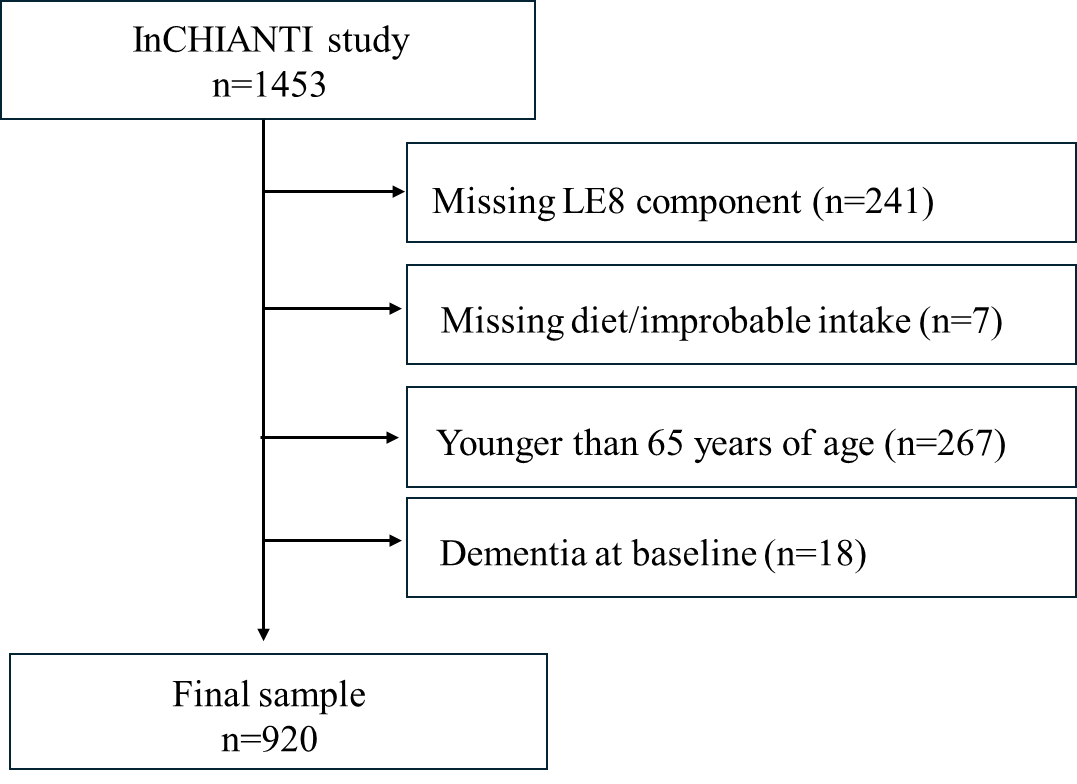
**

**Supplemental Figure 1**. Flow diagram of InCHIANTI study participants.

Supplemental Table 1. Modified LE8 Scoring Criteria^1^

| CVH Category | Method of Data Collection | Scoring |
| --- | --- | --- |
| Diet | Self-reported via Food Frequency Questionnaire (FFQ) | Quantiles of DASH diet adherence (quantified via Mellen’s score^2^)  **Points Quantile**  100 ≥ 95%ile (most ideal diet)  80 75-94%ile  50 50-74%ile  25 25-49%ile  0 1-24%ile (least ideal diet) |
| Physical Activity | Self-reported time spent on physical activity via survey | **Points** **Time and Frequency**  100 Walks > 5 km/day for > 5 days/wk  90 Intense exercise multiple times/wk  80 Moderate exercise > 3 hrs/week  60 Moderate 1-2 hrs or light > 4 hrs/wk  40 Light exercise 2-4 hrs/week  20 Mostly sitting/some walking  0 Hardly any physical activity |
| Smoking Status | Self-reported smoking status | **Points Status**  100 Never smoker  75 Former smoker quit > 5 years ago  50 Former smoker quit > 1 but ≤ 5 years ago  25 Former smoker quit < 1 year  0 Current smoker |
| Sleep Health | Self-reported sleep duration | **Points** **Average # hours of sleep nightly**  100 ≥ 7 but ≤ 9  90 = 9  70 = 6  40 = 5-6 or > 10  20 = 4  0 ≤ 4 |
| BMI | Self-reported weight (kg) and height (m) | **Points Level**  100 <25  70 25.0 - 29.9  30 30.0 - 34.9  15 35.0 - 39.9  0 40.0 |
| Blood Lipids | Non-HDL cholesterol measured through blood tests | **Points Level**  100 < 130  60 ≥ 130 and < 160  40 ≥ 160 and < 190  20 ≥ 190 and < 220  0 ≥ 220  Participants on concurrent cholesterol medication were deducted for an additional 20 points. |
| Blood Glucose | Fasting serum glucose level^3^ | **Points Level (FBG, HbA1c)**  100 FBG < 100 mg/dL  60 FBG ≥ 100 mg/dL and < 126 mg/dL  40 FBG 126 mg/dL and < 154 mg/dL  HbA1c < 7%  30 FBG ≥ 154 mg/dL and < 183 mg/dL  HbA1c = 7-7.9%  20 FBG 183 mg/dL and < 212 mg/dL  HbA1c = 8-8.9%  10 FBG ≥ 212 mg/dL and < 240 mg/dL  HbA1c = 9-9.9%  0 FBG ≥ 240 mg/dL  HbA1c ≥ 10.0%  Participants on concurrent diabetes medication were deducted for an additional 20 points. |
| Blood Pressure | Systolic and Diastolic blood pressure measured by sphygmomanometer with participants in supine position | **Points Level (SBP, DBP)**  100 <120 and < 80 (optimal)  75 120-130 and < 80  50 130-140 and < 90  25 140-160 and < 100  0 ≥ 160 and/or ≥ 100  Participants on concurrent hypertensive medication were deducted for an additional 20 points. |

^1^The criteria for scoring each category implemented in LE8 is based on a published 2022^1^ AHA framework for preserving CVH. The criteria for scoring the categories for smoking, glucose, and physical activity were modified to better suit our study cohort.

^2^ The Mellen’s DASH diet index was used to assess the nutritional value of the diet of our study participants. Adherence to the Mellen score is characterized as the intake of protein, fiber, calcium, magnesium, and potassium, as well as reduced intakes of total fat, saturated fat, cholesterol, and sodium. Assessment of diet in our study was made using quintiles for Mellen scores. For instance, a study participant with a Mellen score falling within the 40th percentile would receive a diet score of 25.

^3^In lieu of HbA1c, fasting serum blood glucose values were obtained from study participants. Using the conversation chart from the American Diabetes Association, we converted the average fasting glucose values of participants to corresponding HbA1c values.
